# Innovating to increase access to diabetes care in Kenya: an evaluation of Novo Nordisk’s base of the pyramid project

**DOI:** 10.1080/16549716.2019.1605704

**Published:** 2019-05-22

**Authors:** Geordan D. Shannon, Hassan Haghparast-Bidgoli, Winnie Chelagat, Joseph Kibachio, Jolene Skordis-Worrall

**Affiliations:** a Centre for Global Health Economics, Institute for Global Health, University College London, London, UK; b Stockholm Environment Institute Africa, Nairobi, Kenya; c Kenyan Ministry of Health, Nairobi, Kenya

**Keywords:** Diabetes, insulin, access, health systems, service delivery

## Abstract

**Background**: The Base of the Pyramid (BoP) project is a public–private partnership initiated by Novo Nordisk that aims to facilitate access to diabetes care for people at the base of the economic pyramid in low- and middle-income countries (LMICs). In Kenya, the BoP, through a partnership model, aims to strengthen five pillars of diabetes care: increased awareness of diabetes; early diagnosis of diabetes; access to quality care by trained professionals; stable and affordable insulin supply; and improved self-management through patient education.

**Objectives**: This study evaluates the extent to which BoP Kenya is scalable and sustainable, whether stakeholders share in its value, and whether BoP Kenya has improved access to diabetes care.

**Method**: The Rapid Assessment Protocol for Insulin Access (RAPIA), an approach developed to provide a broad situational analysis of diabetes care, was used to examine health infrastructure and diabetes care pathways in Kenya. At the national level, the RAPIA was applied in a SWOT analysis of the BoP through in-depth interviews with key stakeholders. At individual and county health system levels, RAPIA was adapted to explore the impact of the BoP on access to diabetes care through a comparison of an intervention and control county.

**Results**: The BoP was implemented in 28 of 47 counties in Kenya. Meru, a county where BoP was implemented, had 35 of 62 facilities (56%) participating in the BoP. Of the five pillars of the BoP, most notable progress was made in achieving the fourth (stable and affordable insulin supply). A price ceiling of 500KSh (US$5) per vial of insulin was established in the intervention county, with greater fluctuation and stock-outs in the non-intervention county. Despite reduced insulin costs, many patients with diabetes could not afford the additive expenses of monitoring, medicines, and travel. Less progress was made over the other pillars, which also faced challenges to sustainability and scalability.

**Conclusion**: In the context of the rising prevalence of non-communicable diseases in LMICs, cross-sector approaches to improving access to care are increasingly needed. Public–private partnerships such as the BoP are necessary but not sufficient to ensure access to health care for people with diabetes at the base of the economic pyramid in Kenya.

## Background

Between 347 and 422 million adults are affected by diabetes around the world [1,2]. The prevalence of diabetes is predicted to rise to 642 million by 2040 and disproportionally affect low- and middle-income countries (LMICs), where 80% of patients are located and where 75% of the burden of mortality from non-communicable diseases (NCDs) occurs [2,3].

In Kenyan adults, the nationally adjusted prevalence of diabetes was estimated to be 3.6% in 2013 and is projected to rise to 4.4% in 2035 [4]. More than 8,700 diabetes-related deaths were registered in Kenya in 2015, almost all under 60 years of age [5]. Diabetes prevalence is higher in urban populations and in men [5], however, most national estimates are derived from adult populations and the prevalence of Type 1 diabetes in children and adolescents is largely unknown [6]. While diabetes is considered under-quantified in this context [7], it is estimated that as many as 60% of people with diabetes in Kenya remain undiagnosed [3]. The number of adults with Type 2 diabetes prescribed insulin in Kenya is also not known, although a household survey performed in Kibera, an informal settlement in Nairobi, estimated that, for those with a formal diagnosis of diabetes, 22.6% used insulin [8]. The rise of NCDs such as diabetes places a double-burden on an already strained health-care system and presents a challenge for the health-care system to respond to the differing needs of both acute and chronic diseases.

Life expectancy and quality of life for people with diabetes can improve, and disease control is possible when diabetes is detected early and managed appropriately [9]. However, if undetected or poorly managed, diabetes can lead to severe and permanent complications including loss of vision, cardiovascular disease, end-stage renal disease, and amputation of the lower extremities [10]. To address this, a basic package of diabetic care should include a healthy diet and physical exercise, blood glucose monitoring, blood pressure control, foot and eye care, and a regular supply of insulin or anti-hyperglycaemic medication if indicated [10]. In LMICs like Kenya, many patients struggle to receive this basic diabetes care. They face several barriers to care that may include distance to the health care facility, lack of awareness, affordability of medicine, availability of diagnostic and monitoring tests, and poor local health system capacity [11,12].

**Figure 1.**
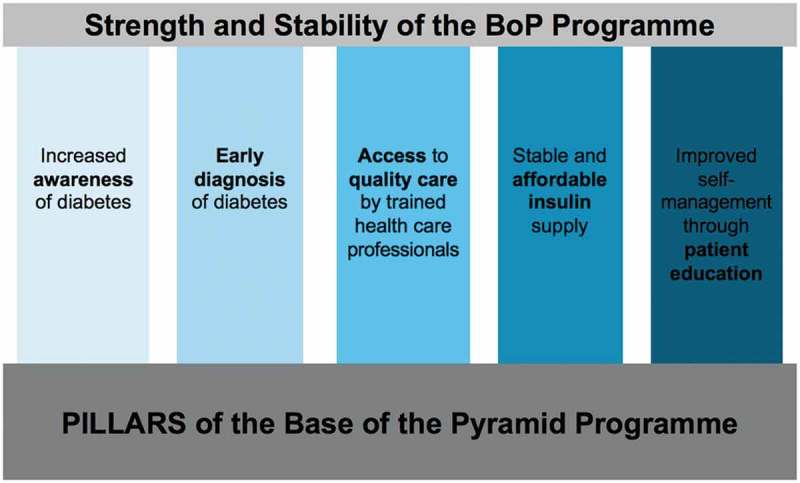
The five pillars of the BoP programme.

To respond to this challenge, the Base of the Pyramid (BoP) project, which is a public–private partnership, aims to facilitate access to diabetes care for the working poor in LMICs [13]. Initiated by Novo Nordisk in 2011, BoP uses a range of different business models adapted to improve health care for people with diabetes at the base of the economic pyramid [14]. In Kenya, the BoP programme’s objective is to bring together stakeholders to ensure: increased awareness of diabetes; early diagnosis of diabetes; access to quality care by trained health-care professionals; stable and affordable insulin supply; and improved self-management through patient education [14]. These are represented symbolically by five pillars which constitute the BoP programme (Figure 1) [15]. The BoP programme works towards Goal 3 of the United Nations Sustainable Development Goals (SDGs), namely ‘to ensure healthy lives and promote well-being for all at all ages’ [16].

With this approach to improving diabetes care in Kenya through a focus on national partnerships and supply-chain strengthening, it was important to assess the impact of the programme holistically. We undertook an independent evaluation of the BoP programme in Kenya to gain insight into its effectiveness, scalability, sustainability, and shared value. The specific objectives of the evaluation were to assess the extent to which BoP Kenya is scalable and sustainable, understand whether all partners share value in the Kenyan BoP scheme, and investigate whether it has improved access to diabetes care.

## Methods

### Setting

With decentralisation of power following the 2010 Kenyan Constitution, Kenya’s 47 Counties each manage their own health systems, including services for NCDs. County health services are arranged into six levels of increasing comprehensiveness. Level 1 are community units that provide community-based care. Level 2 are dispensaries that provide immunisations, basic antenatal care and other primary health care. Level 3 facilities are health centres that are larger than level 1 and 2 facilities and have more comprehensive services, which may involve the dispensation of certain medications and some degree of basic inpatient management. Level 4 are sub-county hospitals, and Level 5 services represent the county referral hospital. Finally, Level 6 services are the national referral hospitals that are located in Nairobi.

Although the Kenyan Constitution declares health to be a universal right [17], progress towards universal health coverage (UHC) in Kenya has been limited [18,19]. A quarter of Kenyan households are further than 8 km away from any sort of health care facility, 23% of Kenyans fail to utilise health care services when they are in need of it and only 25% have some form of health insurance [20]. There was an average life expectancy of 61 (male) and 66 (female) years in 2015 [10]. Out of pocket (OOP) spending as a proportion of total health expenditure decreased from 50% in 2010 to 26% in 2014 but still remains high [21].

NCDs, including diabetes, are increasingly recognised health challenges. The 2015–2020 Kenya National Strategy for the Prevention and Control of NCDs identifies diabetes as one of the four largest chronic disease burdens and has established a roadmap for health promotion, risk factor reduction, and health systems strengthening [22]. Additionally, the National Hospital Insurance Fund (NHIF) recently launched a specific chronic disease package of care [23]. Despite these national initiatives, systems of diabetes care remain inconsistent at the sub-county and county levels, and data on diabetes are severely limited.

### The Kenyan base of the pyramid (BoP) programme

The BoP was designed to address health care for people with diabetes at the ‘base of the economic pyramid’ in Kenya and has been implemented in 28 of 47 counties in Kenya [14.15]. Although Novo Nordisk is a leading insulin producer, the BoP targets wider aspects of diabetes care (Figure 1). Specific programme activities include establishing centres of excellence for diabetes care at local public hospitals, free screening and awareness campaigns, the development of patient education materials in local languages, as well as training of health care professionals, pharmacists and nutritionists in diabetes prevention and care [14]. BoP partners in Kenya include the Kenyan Ministry of Health, County Government Departments of Health, national and local drug distribution networks (Phillips Pharmaceuticals Ltd, Mission for Essential Drugs and Supplies, MEDS), Faith-based organisations (FBOs) including the Kenya Conference of Catholic Bishops (KCCB) and Christian Health Association Kenya (CHAK), and the Kenya Defeat Diabetes Association (KDDA), a national patient advocacy and support network [14]. The BoP specifically works with FBOs who provide primary care at Level 2 and 3 facilities (see *Settings* section, above, for an explanation of levels of care). To limit price mark-ups in the insulin supply chain, while retaining incentives for regular insulin supply, Novo Nordisk signed a memorandum of understanding with every link in the insulin distribution chain, making it difficult for distributors and actors in the value chain to exceed the agreed price of 500 Kenyan Shillings (KSh), or about US$5, per vial.

### Rapid assessment protocol for insulin access (RAPIA)

We adapted the Rapid Assessment Protocol for Insulin Access (RAPIA) in order to evaluate the BoP in Kenya [24]. The RAPIA approach was developed to provide a broad situational analysis of diabetes care, to generate recommendations for health service strengthening to national Ministries of Health and Diabetes Associations. The RAPIA uses a range of approaches to collect information, including interviews, questionnaires, focus group discussions, site visits, and document reviews from various stakeholders [15,24]. This strategy is used to understand the path of insulin and availability of infrastructure, personnel, and resources to diagnose, treat, and care for patients with diabetes and tries to identify barriers that may exist at different levels of the health-care system. RAPIA has the advantage of being both rapid and low-cost, while still yielding data of a high scientific standard. RAPIA has been previously used in Zambia, Mozambique and Mali [25].

The RAPIA collects structured information from the Macro, Meso, and Micro levels of the health system. Macro-level information represents the national level structure of health care services, such as government ministries, national organisations, and other centralised services. Meso-level information is derived from interviews with county health authorities or pharmacies. Micro-level information is collected from individual patients, carers, and service providers [24].

We adapted the RAPIA to the Kenyan health system and tailored it to suit the evaluation of the Base of the Pyramid Project (Appendix 1). At national (Macro) level, the RAPIA was applied in a SWOT analysis of the scalability, sustainability, and shared value in the BoP through 15 stakeholder interviews, national-level process mapping and structured observations in select fields sites. At individual (Micro) and county (Meso) health system levels, RAPIA was adapted to explore the impact of the BoP on access to diabetes care through a comparison of an intervention and control county. We used a combination of in-depth interviews, clinic observations, document reviews and focus group discussions to gather information.

### Comparison of intervention and control counties

For this study, the RAPIA approach was adapted to facilitate comparison between two counties, one with and one without the BoP in Kenya.

The intervention county was selected in a purposive manner from the Kenyan counties in receipt of the programme, by considering firstly if all the components of the BoP programme and integrated diabetes care had been implemented (including patient education, insulin supply chain strengthening, formation of diabetic support groups (DSGs), and staff support systems), and secondly that it was a ‘representative’ Kenyan county. To ensure the representativeness of the county, we analysed geographic, demographic, health and economic indicators of all counties included in the BoP. We selected Meru county as our intervention county based on this analysis and reassurance from the BoP management team that all components of the programme were implemented. This county was then purposively matched to a comparison or control county based on the same geographic, demographic, health and economic indicators, in which the BoP was not implemented. Based on this process, Trans Nzoia was selected as the control county. The study sites are detailed in Table 1.

**Table 1.** County summary of Meru and Trans Nzoia.

Indicator	Meru	Trans Nzoia
Location	300 km northeast of Nairobi	390 km northwest of Nairobi
Population	1,584,575	956,559
% of population located in urban areas	16%	20%
Gross domestic product (GDP) per capita	US$533	US$349
GINI index	0.348	0.360
Educational status	18% secondary or above 62% primary education only 21% no formal education	21% secondary or above 59% primary education only 20% no formal education
Health system overview	116 public facilities, 66 FBOs, 3 non-government organisations, and 20 private sector owned facilities. 58% of all services are run through the government	54 public facilities, 15 FBOs, 5 non-government organisations and 78 private sector owned facilities, generally small private clinics

Data sources [26–29].

### Data collection

At the national (Macro) level, we performed a total of fifteen in-depth interviews with key stakeholders (Table 2). In addition, we undertook structured observations at the Nakuru Centre of Excellence Launch, the World Diabetes Day conference in Nairobi, and with each of the seven national BoP partners. Macro-level interviews established acceptability, scalability, and sustainability of the BoP in Kenya.

**Table 2.** Summary of Macro-, Meso-, and Micro-level interviews.
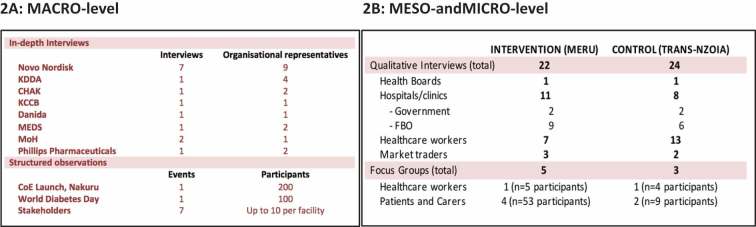

KDDA: Kenyan Defeat Diabetes Association; CHAK: Christian Health Association Kenya; FBO: Faith-based organisation; KCCB: Kenyan Conference of Catholic Bishops; MEDS: Mission for Essential Drugs and Supplies; MoH: Ministry of Health; CoE: Centre of Excellence.

In Meru, the intervention county, a total of 22 in-depth interviews and five focus group discussions (FGDs) were conducted (Table 2). In the control county, Trans Nzoia, a total of 24 in-depth interviews and three FGDs were conducted. Data were collected from patients with diabetes, medical staff, community health workers, pharmacists, lab technicians and health service administrators. The Meso- and Micro-level interviews explored sustainability and value sharing, while also describing barriers to care and the effect of the BoP on patient care. In addition, at Meso and Micro levels, data on the five core modalities of BoP including increased diabetes awareness, early diagnosis, access to care, stable and affordable insulin supply and improved patient management, were collected.

Where possible and appropriate, data from qualitative key informant interviews were supplemented with available secondary data provided by Novo Nordisk. Secondary data from Novo Nordisk included information on the number of clinics in operation, as well as the nature and quantity of products and services supplied. The research team established a clear division between Novo Nordisk and the research process to reduce bias and minimise contaminations.

### Research access and ethics

Data collection occurred between October and December 2016. UCL partnered with the Stockholm Environment Institute Africa (SEI-Africa), a not-for-profit research agency based in Nairobi, for local expertise and research support. The Kenyan Novo Nordisk Office facilitated field research and interviews with all Macro-level stakeholders. Novo Nordisk also assisted in setting-up Meso- and Micro-level interviews in Meru county. Researchers from SEI-Africa and central FBO facility coordinators helped in making introductions locally in Trans Nzoia. All participants were recruited through local health services and consented to their involvement in the interview or focus group discussion process, including the use of audio-visual recordings. Researchers maintained independence from Novo Nordisk throughout the evaluation, and fieldwork and findings were not shared with Novo Nordisk until the conclusion of the study.

Ethical approval for the research was granted by the UCL Research Ethics Committee (5406/002) and the Maseno University Ethics Committee (00334/16).

### Interviews and analysis

A team of three people (supervisor, interviewer/translator and note-taker) conducted all field interviews. We performed the interviews in either Kiswahili or English. Interviews with stakeholders, staff and officials were performed in English, while interviews with patients, carers and merchants were performed in Kiswahili. After obtaining consent, all interviews were tape-recorded and detailed interview notes taken. Data collection and analysis took place simultaneously, and an initial analysis of each interview was made before the next interview. This allowed modification of the interview guides for the next interview if important issues emerged. Interview notes were thematically analysed, and a sample of the tape recordings was transcribed and translated by a member of the team in order to triangulate and validate the findings from the interview notes.

## Results

Our key findings are presented in terms of the five pillars of the BoP: increased awareness of diabetes; early diagnosis of diabetes; access to quality care by trained health-care professionals; stable and affordable insulin supply; and improved self-management through patient education.

### Awareness of diabetes

At a national level, Novo Nordisk activities aimed to encourage awareness of diabetes and engagement in diabetes care. Their reported focus was on stakeholder engagement, networking across sectors, and specific educational activities. BoP partners also attempted to raise awareness of diabetes through annual education days or education campaigns. However, at Meru County level, there were limited BoP outreach activities which resulted in low awareness of diabetes and minimal understanding of the BoP and Novo Nordisk. There was local enthusiasm for such outreach activities and this represents a future opportunity. Although BoP-related outreach and education activities were not present in Trans Nzoia, there was an annual march organised around World Diabetes Day to encourage greater awareness and education about diabetes and to encourage screening. This was coordinated by the Kitale DSG alongside the main government referral hospital in Kitale. We also observed non-BoP community health volunteer models of outreach operating in both Trans Nzoia and Meru; this approach was identified by interview participants as favourable for the local context.

### Early diagnosis

In Meru, the BoP facilitated some free screening activities at Level 4 FBO facilities between 2013 and 2015, involving free finger stick blood glucose testing with a glucose meter along with education and free tea. This campaign was perceived by staff as being successful in attracting patients to health care facilities and improving initial contact with the health-care system. However, these campaigns were reportedly short-lived. Funding for these activities has since ceased, and some FBO facilities felt a sense of disappointment and frustration that the costs of screening were now passed to the patient and that, subsequently, less people accessed screening and early diagnosis. Furthermore, access to regular blood glucose level (BGL) testing with a finger stick glucose meter – as the most routinely used tests to guide both diagnosis and management of diabetes – were perceived to be unaffordable on a regular basis. Thus, many patients were being managed without a good sense of their BSL profile. One practitioner described it as ‘like driving a car in the dark without the lights on.’

There were no coordinated community screening or early diagnosis activities in place in Trans Nzoia. This meant that people risked presenting in later stages of their disease and with advanced complications, placing higher burden on the health-care system through increased intensity of care and resources. In both locations, diabetes groups also felt that regular BSL testing was unrealistic and too expensive to access on a routine basis as required for adequate diabetes management.

### Access to quality care by trained health-care professionals

Barriers to access were evaluated from patient perspectives in both counties. Poverty, geography, education, gender, and health system limitations were barriers to accessing appropriate diabetes care. The combined costs of seeking health care were often insurmountable and were identified as one of the most prominent issues in obtaining adequate health care. Costs involved transport to clinics, supplementary equipment costs (such as syringes and glucose meters), providing healthy food, opportunity costs of missing a day of work, upfront consultation and diagnostic fees, and medication costs. Patients reported they were unable to afford essential medicines because of poverty, and trade-offs in household financial decisions between food, education, transport, and medicine were common. One commonly reported behaviour was that patients would either not use insulin when medically indicated or would part-treat themselves with insulin in order to make it last longer. Improved affordability of insulin was therefore not sufficient to overcome additive costs of accessing care.

Through the BoP programme, some health care professionals in Meru received training in the form of printed resources and annual educational activities. The printed materials were greatly valued by both patients and staff and were in high demand. Training was offered annually to only one or two staff per facility, meaning that the programme did not cover the demand for education. In addition, the training events took place in centrally located venues, meaning that participants had to travel long distances. In the control county, staff did not have access to formal educational opportunities, however, one motivated staff member at a Trans Nzoia FBO lobbied Novo Nordisk to provide access to a range of printed educational resources which they informally distributed to their network. An additional challenge to health worker capacity-building in both locations was the reportedly high staff turnover rates, where recipients of BoP training left for other facilities. This translated into low awareness of the BoP programme and lower levels of diabetes knowledge in rural facilities. The mentoring component of the BoP education package was seen as valuable by those involved but needed expansion to ensure all rural staff are linked to greater support and expertise.

### Stable and affordable insulin supply

In Meru county, a more consistent supply of insulin was identified: one unit of Mixtard® (a suspension containing both fast-acting – soluble – and intermediate-acting – isophane – insulin) was being sold at a price of 500 KSh (about US$5). This stability in supply has effectively established a price ceiling of 500–600 KSh for Mixtard® insulin both within participating facilities and in surrounding markets and private pharmacies in Meru, where vials were previously sold for up to 1,800 KSh (about US$18) (Table 3). In Trans Nzoia, where the BoP was not formally running, there was comparatively greater fluctuation in the price of a vial of insulin. Insulin was occasionally available more affordably at government clinics in both locations, where the subsidised price was 200 KSh (about US$2). However, insulin at this price and in these clinics was found to be frequently out of stock, meaning that patients had to purchase it privately and at much higher prices than the price ceiling in BoP areas.

**Table 3.** Direct medical and non-medical costs involved in seeking care for diabetes.

Comparative costs of diabetes care in Meru and Trans Nzoia							
		Meru (intervention)			Trans Nzoia (control)		
		Govt	FBO	Private	Govt	FBO	Private
Medicine	Insulin	Buy at 320 KSh and sell at 200 KSh/vial as per govt directive	500 KSh/vial or 50 KSh/unit as inpatient	Street cost 550–600/vial (previously up to 1800 KSh)	Buy at 320 KSh and sell at 200 KSh/vial but often out of stock	Not available	Price fluctuates from 500–2200 KSh/vial (only available in Kitale city)
	AHGs	2–4 KSh per tablet (Metformin)	50 KSh per month	5–6 KSh per tablet	Metformin 2 KSh/500 mg tablet Glibeclamide 2 KSh/tablet currently out of stock	Not available	5–6 KSh per tablet
	Medications for comorbidities	-	-	-	Aspirin 75 mg 1 KSh/tab Enalapril 2 KSh/tab vit B 2 KSh/tab	-	-
Equipment	Glucose meter	Free with purchasing strips	-	3000–4000 KSh	Free with strips	-	3000–4000 KSh
	Strips	1500 KSh for 50 strips	100 KSh per test	-	-	100 KSh per test	-
	HbA1C machine	Upto 10,000 KSh for strips plus machine	-	-	-	-	-
	Syringes	-	15 KSh/syringe	-	-	-	-
Diet		Variable, but agreed across all locations that nutritious, fresh food more expensive ‘Cash crops’ sold at market, diet mainly maize					
Travel		Up to 2000 KSh per month of travel			300–2000 per month on travel		
Medical consultations		200/consultation	150–200				
Lab testing	HbA1C	1000 KSh/test	400–700 KSh		600 KSh/test		
	BSL	150 KSh/test	50–140 KSh		100–120 KSh/test		

Figures triangulated between interviews, focus group discussions, and field observations.

Given the high rates of government stock-outs of insulin and other medicines, consistency in supply was highly valued by the interviewed patients.

Despite more consistently reduced insulin costs in the BoP areas, many patients with diabetes could not afford the combined direct expenses of regular monitoring, testing, medical consultations and medicine. Direct non-medical expenses like the costs of travelling and also lost wages (or opportunity cost) of care seeking, further increased the financial burden of diabetes care (Table 3). Average GDP per capita in Meru was US$533 (US$1.51/day), and Trans Nzoia was US$349 (US$0.99/day), meaning that the average person in these counties lives below the poverty line of US$1.90 per day. For many who were living at or below the poverty line, the costs relating to seeking medical care were often insurmountable. Patients interviewed experienced competing financial priorities forcing a difficult choice between food, living expenses, education, and essential health care.

### Self-management through patient education

To address some barriers to diabetes care, multiple DSGs were established in Meru by both FBOs (BoP) and the government (non-BoP), which took normally place on the diabetes clinic days. These groups encouraged patients to share experiences and strategies to cope with diabetes. However, DSGs ran on limited resources and relied on individual members taking time to attend the group. Groups felt under-supported and under-resourced and thus were unable to provide the optimal level of support for their members. In addition, the price of consumables for diabetes care, from glucose meter strips to syringes, were seen as additive expenses that were out of reach for many. DSGs suggested models of support via joint purchasing of glucose meter units and strips to share around the patient group.

## Discussion

The objectives of this study were to explore whether the Kenyan BoP scheme is sustainable, understand whether all partners share value in the BoP, assess the extent to which BoP is scalable, and investigate whether BoP has improved access to diabetes care. We explored these areas over the five pillars of the BoP, above.

### Sustainability, scalability, shared value, and access to diabetes care

One of the main focal points of **sustainability** of the BoP model was the price point of insulin, which was seen as a balance between charity and profit, and as a way to ensure the financial durability of the BoP. The lower price of insulin from national suppliers led to an increase in orders placed at national level; however, it has not yet translated into a financially sustainable model for Novo Nordisk. Despite this, a secure system of insulin ordering, procurement, and supply now exists as a result of BoP.

At the national level, all partners had a strong sense of **shared value** in BoP. Despite reports that national BoP partner organisations felt stretched financially to provide services (insulin, staff education or staff support), they felt that the service they provided was an important one. However, local health care providers felt left behind and require further support to ensure they, too, feel a sense of ownership and shared value in the programme.

In terms of **scale**, the BoP may, in fact, benefit from more focused activities at the county-level to enhance overall effectiveness. Despite strong Macro-level activities, many of our findings suggest that front-line staff and patients do not benefit from the full potential of the BoP. Therefore, prior to expansion of the programme, greater focus on strengthening existing components is necessary. The impact of the BoP on national insulin supply chains may offer some benefit to neighbouring countries through partners such as MEDS who supply to East Africa. At the micro level, because of the nature of human movement and small-scale business in Kenya, the lower price of insulin in BoP counties such as Meru may also have a natural flow-over effect into non-intervention counties.

Of all the factors influencing **access to care**, we found that the consistency and availability of insulin, along with a price ceiling of around US$5 for a vial of insulin, were valued in the context of high stock-outs and price fluctuations in government health services. However, access to affordable insulin was only one piece of a larger and more complex puzzle. The additive costs of seeking medical treatment meant that many still could not afford adequate diabetes care. In the context of high rates of poverty in rural Kenya, many individuals and households faced difficult choices about spending the limited resources they had. For many, this meant that their diabetic care was severely compromised.

Out-of-pocket health expenditure (expressed as % of current health expenditure) in Kenya in 2015 was 33% [30] This means that in a large number of cases, individuals pay for health care from limited personal savings. This scenario places households at risk of catastrophic health spending and subsequent impoverishment. The average Kenyan household spends over a tenth of their annual budget on health care payments, however, this is higher for the poorest of Kenyan households who spend a third of their resources on health care payments [30]. About 1.3% of Kenyans are pushed below the international poverty line annually because of health care payments [19,30]. The BoP has the opportunity to alleviate some of this burden through the provision of quality and affordable care, through strengthening the pillars of the BoP beyond insulin provision (awareness, diagnosis, quality of care). In the context of Universal Health Coverage and the SDGs goal of equality of access to health care, it is important to improve components and systems of health care for NCDs such as diabetes.

### Strengths, weaknesses, opportunities, and threats

The main strengths of the BoP included networking and stakeholder engagement through Macro-level educational activities and partnerships. The BoP programme was the first of its kind in Kenya and has initiated a network of action around diabetes care and has contributed to opening up the conversation around NCDs at a national level. Another strength of the BoP is the ability to price-regulate vials of insulin to 500KSh at the consumer level. This has ensured a more consistent demand and supply cycle.

However, in comparison with other ongoing front-line programmes, the BoP initiative is relatively unknown among patients and lower level health workers. Although it is not a priority of the programme, some element of ‘branding’ at the patient and service provider level could ensure the messages and impact of the programme are better known and more effectively taken-up at the micro- and meso-level. Despite a very strong macro-level approach to insulin provision and public–private partnerships, the micro- and meso-level activities relating to the five pillars of the BoP need to be strengthened to maximise overall programme effectiveness. With the view of transitioning towards UHC, there are many issues around infrastructure and sustainability to address in conjunction with county governments and the Ministry of Health.

As with many LMICs, data availability, and data systems are significant limiting factors in research on NCDs in Kenya. At present, we have very limited information on the prevalence of diabetes at county or sub-county levels. Data limitations in Meru and Trans-Nzoia counties made a quantitative evaluation of the impact of the BoP programme impossible. The BoP, as a coordinated and integrated programme with a wide reach into rural and under-resourced areas in Kenya, has the opportunity to assist with the development and strengthening of data collecting systems. This would not only be beneficial to the evaluation of the BoP project itself but also would benefit diabetes and NCD research in Kenya and beyond. This project has the opportunity to encourage the strengthening of local and national-level health management information systems.

There is an opportunity to collaborate with interested partners in the delivery of a more holistic NCD package. As identified above, a community health volunteer model of outreach has the capacity to increase community awareness and diagnosis and would be an excellent model for collaboration. Novo Nordisk has purposively taken the approach of partnering with a range of existing organisations to work within existing national structures. The BoP has focused on existing networks of FBOs around the country, who have substantial rural coverage. A lot of the network building has been at a national level, which has meant there has been limited contact with BoP activities ‘on the ground.’ There is the potential to work closer with FBO partners and/or DSGs to ensure greater support and capacitation of staff and patients on the ground to enhance diabetic care.

A macro-level threat underlying the BoP activities was the existence of multiple competing health care priorities in Kenya, in addition to the historical legacy of vertical programmes targeting singular communicable diseases. The Kenyan Ministry of Health’s Department of Non-Communicable Diseases is driving increased focus on a number of NCDs and their inter-relations, as well as coordinating multiple nongovernmental and governmental programmes. A recent increase in national attention towards NCDs has culminated in the publication of the Kenya National Strategy for the Prevention and Control of Non-Communicable Diseases, 2015–2020 [32]. The BoP has taken advantage of the willingness expressed in the national NCD strategy of embracing partnerships and utilising private sector capacity. But there are many more opportunities for the BoP to continue to expand the scope and reach of its work.

### Research limitations and next steps

Our research was limited in the following ways. First, the evaluation above occurred at the 5-year mark of the BoP in Kenya. Embedding evaluation approaches into the project planning cycle can lead to a greater understanding of the process of change as well as the overall impact of the intervention. Second, the limited quantitative data availability meant that we relied heavily on fact-finding and qualitative interviews. Third, the budget and timescales enabled only the RAPIA approach, which is suitable for a rapid situational analysis and systematic overview, rather than an in-depth evaluation. The strength of the RAPIA, aside from its fast timescale, was its agility to be adapted to the Kenya context, as well as to position the impact of the BoP within the levels ofKenya’s health system. Fourth, because of the scope of the evaluation, we were only able to select two counties (an evaluation and control county) to compare, meaning we only captured a snapshot of the complexities of diabetes care in Kenya. Finally, while every effort was made to reduce the influence of Novo-Nordisk on the findings, we cannot entirely rule out the risk of positive bias.

At the Macro level, possible next steps for the BoP would be to continue to align with the Ministry of Health work plan and to expand a sense of national ownership, not simply to operate as a stand-alone project. Further, BoP Kenya may consider more strategic partnerships along to the pathway of diabetic care, including suppliers of point of care diagnostics. At the Meso Level, investment in health system infrastructure such as diagnostic equipment and human resources is critical; in particular, a focus on health worker education and staff satisfaction could ensure greater staff retention and continuity of care. At the Micro level, increased community awareness is possible, but requires sustained efforts on behalf of the BoP partners. This may include the enhanced role of the peer mentor and diabetic support group activities. Beyond awareness and education, there is an opportunity to enhance diagnosis through ensuring diagnostic equipment is reliably available closer to the community.

## Conclusion

In the context of rising prevalence of NCDs in LMICs, improving access to diabetes care is essential. BoP demonstrates that collaborative approaches to improving access to diabetes care have the potential for improving diabetes care, but require sustained efforts at all levels of the health system and consideration of barriers to care from patients’ perspectives. Of the five pillars of the BoP, most notable progress was made in achieving the fourth pillar (stable and affordable insulin supply). Less progress was made over the other pillars, which also faced challenges to sustainability and scalability.

At the macro level, the BoP engaged stakeholders at a national level to ensure structural change, but needs to invest more in local approaches to overcoming barriers to care, which may include supporting local DSGs, enhancing self-monitoring with glucometers, community outreach, and greater capacity-building in lower-level health centres. Our evaluation also highlights priority areas for further improvement including further reducing the overall financial burden of diabetes care, scaling up the BoP to other counties in Kenya and improving the visibility of the education, awareness and screening programmes.

## Paper context

With rising NCD prevalence in LMICs, cross-sector approaches to improving access to diabetes care are needed. We adapted the Rapid Assessment Protocol for Insulin Access (RAPIA) to evaluate the impact of the BoP, a public–private partnership that aims to facilitate access to diabetes care in Kenya. The BoP engaged stakeholders at a national level to ensure structural change but needs to invest more in local approaches to overcoming barriers to care.

## Appendix Appendix 1.

**Table A1.** Adapting the RAPIA method to the Kenyan BoP Evaluation.

Stakeholder	RAPIA interview themes	Objectives addressed
*MACRO-LEVEL INTERVIEWS*		
Novo-Nordisk	*Distribution of insulin* *Insulin tendering and purchase* *Value share to Novo-Nordisk* *Perceptions of BoP acceptability, effectiveness and sustainability* *Stakeholder engagement in BoP* *Institutional support for sustained and up-scaled BoP activities*	Is BoP **scalable**? Is BoP **sustainable**? Is there **shared value** in the scheme?
Phillips Pharmaceuticals Ltd	*Distribution of insulin* *Insulin tendering and purchase* *Value share to Phillips* *Awareness of BoP* *Perceptions of BoP acceptability, effectiveness and sustainability* *Stakeholder engagement in BoP* *Institutional support for sustained and up-scaled BoP activities*	Is BoP **scalable**? Is BoP **sustainable**? Is there **shared value** in the scheme?
Ministry of Health	*Organisation of diabetes care* *Resources for diabetes care* *National diabetes programmes* *Pricing of insulin* *Distribution of insulin* *Funding for insulin and diabetes* *Insulin tendering and purchase* *Educating patients* *Awareness of BoP* *Perceptions of BoP acceptability, effectiveness and sustainability* *Stakeholder engagement in BoP* *Institutional support for sustained and up-scaled BoP activities*	Is BoP **scalable**? Is BoP **sustainable**? Has BoP **improved access** to diabetes care?
Ministry of Trade	*Trade issues (laws, barriers)* *Trade infrastructure* *Awareness of BoP* *Perceptions of BoP acceptability, effectiveness and sustainability* *Stakeholder engagement in BoP* *Institutional support for sustained and up-scaled BoP activities*	Is BoP **scalable**? Is BoP **sustainable**? Has BoP **improved access** to diabetes care?
Ministry of Finance	*Funding of health system* *Taxes on insulin* *Funding for insulin and diabetes* *Awareness of BoP* *Perceptions of BoP acceptability, effectiveness and sustainability* *Stakeholder engagement in BoP* *Institutional support for sustained and up-scaled BoP activities*	Is BoP **scalable**? Is BoP **sustainable**? Has BoP **improved access** to diabetes care?
Local drug distribution networks (e.g. MEDS)	*Pricing of insulin* *Distribution of insulin* *Awareness of BoP* *Perceptions of BoP acceptability, effectiveness and sustainability* *Stakeholder engagement in BoP* *Institutional support for sustained and up-scaled BoP activities* *Perceived value share from BoP*	Is BoP **sustainable**? Is there **shared value** in the scheme? Has BoP **improved access** to diabetes care?
Faith-based associations, e.g. The Christian Health Association of Kenya (CHAK) and the Kenya Conference of Catholic Bishops (KCCB)	*Organisation of diabetes care* *Resources for diabetes and insulin* *Distribution of insulin* *Awareness of BoP* *Perceptions of BoP acceptability, effectiveness and sustainability* *Stakeholder engagement in BoP* *Institutional support for sustained and up-scaled BoP activities* *Perceived value share from BoP*	Is BoP **sustainable**? Is there **shared value** in the scheme? Has BoP **improved access** to diabetes care?
Diabetes Associations including Kenya Defeat Diabetes Association and Diabetes Kenya Association	*Issues with diabetes and insulin* *Education campaigns* *Awareness of BoP* *Perceptions of BoP acceptability, effectiveness and sustainability* *Stakeholder engagement in BoP* *Institutional support for sustained and up-scaled BoP activities* *Perceived value share from BoP*	Is BoP **sustainable**? Is there **shared value** in the scheme? Has BoP **improved access** to diabetes care?
Central Medical Store/Kenya Medical Supplies Authority (KEMSA)	*Insulin tendering and purchase* *Insulin distribution and storage* *Insulin pricing* *Awareness of BoP* *Perceptions of BoP acceptability, effectiveness and sustainability* *Stakeholder engagement in BoP* *Institutional support for sustained and up-scaled BoP activities*	Is BoP **sustainable**? Is there **shared value** in the scheme? Has BoP **improved access** to diabetes care?
*MESO-LEVEL INTERVIEWS*		
County/district health organisation	*Issues with diabetes and insulin* *Organisation of diabetes care* *Awareness of BoP* *Perceptions of BoP acceptability, effectiveness and sustainability* *Stakeholder engagement in BoP* *Institutional support for up-scaled BoP*	Is BoP **scalable**? Is BoP **sustainable**? Is there **shared value** in the scheme? Has BoP **improved access** to diabetes care?
Hospitals, clinics, health centres, dispensaries, etc.	*Treatment and management of diabetes* *Access to appropriate tools to diagnose and treat patients* *Infrastructure present and/or lacking for insulin provision* *Awareness of BoP* *Perceptions of BoP acceptability, effectiveness and sustainability* *Stakeholder engagement in BoP* *Institutional support for up-scaled BoP*	Is BoP **sustainable**? Is there **shared value** in the scheme? Has BoP **improved access** to diabetes care?
Laboratories	*Infrastructure for diagnosis and follow-up* *Awareness of BoP* *Perceptions of BoP acceptability, effectiveness and sustainability* *Institutional support for up-scaled BoP*	Is BoP **sustainable**? Has BoP **improved access** to diabetes care?
Pharmacies	*Insulin distribution and storage* *Insulin pricing* *Awareness of BoP* *Perceptions of BoP acceptability, effectiveness and sustainability* *Stakeholder engagement in BoP* *Institutional support for up-scaled BoP*	Is BoP **sustainable**? Has BoP **improved access** to diabetes care?
*MICRO-LEVEL INTERVIEWS*		
Health workers and traditional healers	*Problems encountered in diagnosis and treatment of patients* *Training* *Infrastructure present and/or lacking* *Tools present and/or lacking* *Awareness of BoP* *Perceptions of BoP acceptability, effectiveness and sustainability* *Stakeholder engagement in BoP* *Institutional support for up-scaled BoP*	Is BoP **sustainable**? Has BoP **improved access** to diabetes care?
Market traders selling drugs	*Awareness of diabetes and Insulin* *Access to insulin for resale* *Pricing of insulin* *Awareness of BoP* *Perceptions of BoP acceptability, effectiveness and sustainability* *Stakeholder engagement in BoP* *Institutional support for up-scaled BoP*	Is BoP **sustainable**? Has BoP **improved access** to diabetes care?
Patients and carers	*Diagnosis* *Access to treatment* *Cost of treatment* *Awareness of diabetes* *Awareness of BoP* *Perceptions of BoP acceptability, effectiveness and sustainability*	Is BoP **sustainable**? Has BoP **improved access** to diabetes care?
